# Prostanoid EP_2_ Receptors Are Up-Regulated in Human Pulmonary Arterial Hypertension: A Key Anti-Proliferative Target for Treprostinil in Smooth Muscle Cells

**DOI:** 10.3390/ijms19082372

**Published:** 2018-08-12

**Authors:** Jigisha A. Patel, Lei Shen, Susan M. Hall, Chabha Benyahia, Xavier Norel, Robin J. McAnulty, Shahin Moledina, Adam M. Silverstein, Brendan J. Whittle, Lucie H. Clapp

**Affiliations:** 1 Institute of Cardiovascular Science, University College London, London WC1E 6JF, UK; jigisha.a.patel@ucl.ac.uk (J.A.P.); lei.shen.09@ucl.ac.uk (L.S.); 2 Infectious Diseases and Immunity, University College London, London WC1N 1EH, UK; mandshall@btinternet.com; 3 INSERM U1148, CHU X. Bichat, Paris Cedex 18, 75877 Paris, France; chabhabio@hotmail.fr (C.B.); xnorel@hotmail.com (X.N.); 4 Respiratory Centre for Inflammation and Tissue Repair, University College London, London WC1E 6JF, UK; r.mcanulty@ucl.ac.uk; 5 Paediatric Cardiology, Great Ormond Street Hospital, London WC1N 3JH, UK; Shahin.Moledina@gosh.nhs.uk; 6 United Therapeutics Corporation, Research Triangle Park, NC 27709, USA; asilverstein@unither.com; 7 William Harvey Research Institute, Queen Mary University of London, London EC1M 6BQ, UK; b.j.whittle@qmul.ac.uk

**Keywords:** prostacyclin, prostaglandin, EP_2_ receptor, human, treprostinil, selexipag, pulmonary arterial smooth muscle cell proliferation, IP receptor agonists, pulmonary hypertension

## Abstract

Prostacyclins are extensively used to treat pulmonary arterial hypertension (PAH), a life-threatening disease involving the progressive thickening of small pulmonary arteries. Although these agents are considered to act therapeutically via the prostanoid IP receptor, treprostinil is the only prostacyclin mimetic that potently binds to the prostanoid EP_2_ receptor, the role of which is unknown in PAH. We hypothesised that EP_2_ receptors contribute to the anti-proliferative effects of treprostinil in human pulmonary arterial smooth muscle cells (PASMCs), contrasting with selexipag, a non-prostanoid selective IP agonist. Human PASMCs from PAH patients were used to assess prostanoid receptor expression, cell proliferation, and cyclic adenosine monophosphate (cAMP) levels following the addition of agonists, antagonists or EP_2_ receptor small interfering RNAs (siRNAs). Immunohistochemical staining was performed in lung sections from control and PAH patients. We demonstrate using selective IP (RO1138452) and EP_2_ (PF-04418948) antagonists that the anti-proliferative actions of treprostinil depend largely on EP_2_ receptors rather than IP receptors, unlike MRE-269 (selexipag-active metabolite). Likewise, EP_2_ receptor knockdown selectively reduced the functional responses to treprostinil but not MRE-269. Furthermore, EP_2_ receptor levels were enhanced in human PASMCs and in lung sections from PAH patients compared to controls. Thus, EP_2_ receptors represent a novel therapeutic target for treprostinil, highlighting key pharmacological differences between prostacyclin mimetics used in PAH.

## 1. Introduction

Pulmonary arterial hypertension (PAH) is a highly proliferative, vascular remodelling disease leading to right heart failure and death, with endothelin-1 (ET-1) implicated as an important mediator of vasoconstriction and remodelling in this disease [1,2]. Prostacyclin and its chemically stable analogues, iloprost and treprostinil, are used extensively in the treatment of PAH [2,3,4]. Early work on prostacyclin or its analogues (the prostacyclins) considered that activity at the prostanoid IP receptor significantly contributed to their pharmacological properties in humans [5], including potent vasodilator effects in the pulmonary vasculature [2,6,7] and anti-proliferative effects in distal pulmonary arterial smooth muscle cells (PASMCs) derived from normal lungs [8,9]. Based on this concept, selexipag, a novel non-prostanoid and highly selective IP receptor agonist was developed for PAH [10,11] and is now a clinically approved treatment [12].

Prostacyclins have diverse effects on prostanoid IP, EP_1_, EP_2_, EP_3_ or DP_1_ receptors at clinical concentrations [5]. Radioligand binding assays for human prostanoid receptors showed that treprostinil had high affinity towards EP_2_, DP_1_ and IP receptors [13], and this was more recently independently confirmed in several isolated smooth muscle preparations [14]. Furthermore, compared to other prostacyclin analogues, enhanced and more prolonged cyclic adenosine monophosphate (cAMP) generation was previously reported for treprostinil in both human PASMCs (HPASMCs) and mouse alveolar macrophages, strongly suggesting signalling through additional Gs-coupled receptors [9,15]. In macrophages this was largely accounted for by the activation of EP_2_ receptors [15]. That other receptors might contribute to the action of treprostinil in the pulmonary vasculature, is supported by our previous work where IP receptor-independent mechanisms largely mediated the anti-proliferative effects of treprostinil in HPASMCs derived from PAH patients [16]; this occurred against a backdrop of decreased IP receptor expression.

The role of EP_2_ receptors in regulating pulmonary smooth muscle proliferation has yet to be established. However, these receptors underlie the anti-proliferative effects of prostaglandin E_2_ in airway smooth muscle cells [17], they are upregulated by smooth muscle growth factors known to be increased in PAH [18,19,20] and are protective against neointimal hyperplasia caused by vascular injury [20]. By contrast, there are no reports so far showing DP_1_ receptors significantly regulating smooth muscle proliferation.

Here, we hypothesize that treprostinil exerts strong anti-proliferative actions through the activation of the EP_2_ receptor in HPASMCs derived from patients with PAH, which becomes the dominant pharmacological target either because of the enhanced expression of EP_2_ receptors and/or the down-regulation of IP receptors. Moreover, treprostinil has a 10 fold higher affinity at the EP_2_ receptor compared to the IP receptor [13]. In the present work, we defined the role of IP and EP_2_ prostanoid receptors using highly selective prostanoid receptor agonists, antagonists, and gene-silencing techniques using small interfering RNAs (siRNAs) to “knockdown” the EP_2_ receptor. The effects of treprostinil were directly compared to the active selexipag metabolite, MRE-269 (ACT-333679), a specific agonist at the IP receptor [10], whose activity in HPASMCs from PAH patients has not been examined previously. EP_2_ receptor expression was assessed in human pulmonary vascular tissue from normal and PAH patients using quantitative-PCR (qPCR) and immunohistochemical techniques. This work now demonstrates that the selexipag metabolite acts exclusively via the IP receptor to modulate the proliferation of smooth muscle cells. By contrast, this study identifies for the first time, that EP_2_ receptors are upregulated in PAH and are important negative modulators of pulmonary artery smooth muscle proliferation, thus representing a previously unrecognized therapeutic target for treprostinil.

## 2. Results

### 2.1. Patient Characteristics

Patients were classified according to updated clinical guidelines for pulmonary hypertension [21] and had a mean age of 15.1 ± 6.2 year (yr), a mean pulmonary artery pressure of 72 ± 5.2 mmHg (*n* = 9) and a pulmonary vascular resistance index (PVRI) of ≥19 Wood units.m^2^ (Table S1). Samples were obtained from patients (*n* = 10) diagnosed as having idiopathic PAH (IPAH) who went on to have a transplant after failed treatment or who had died. However, on clinical examination at the time of transplant, 6 patients had other complications confirmed, including 5 patients with PAH associated with minor heart defects. All patients were treated with bosentan and a prostacyclin, with 5 also treated with sildenafil (mean duration of 2.7, 2.8 and 3.5 yr, respectively). Gross pathological changes in the lungs can be seen in Figure S1. Histological staining with hematoxylin and eosin (H&E; left panel), as well as with Van Gieson (EVG; right panel), showed gross structural changes in lung sections from patients with PAH. Small arteries were more muscularised compared to sections from normal lungs, and an increased in collagen deposition was observed (Figure S1). Both haemodynamic and histological changes reported in the patient group of the study are consistent with a clinical classification of group 1 pulmonary arterial hypertension with end-stage disease.

### 2.2. Anti-Proliferative Activity of Treprostinil and MRE-269

**Figure 1:**
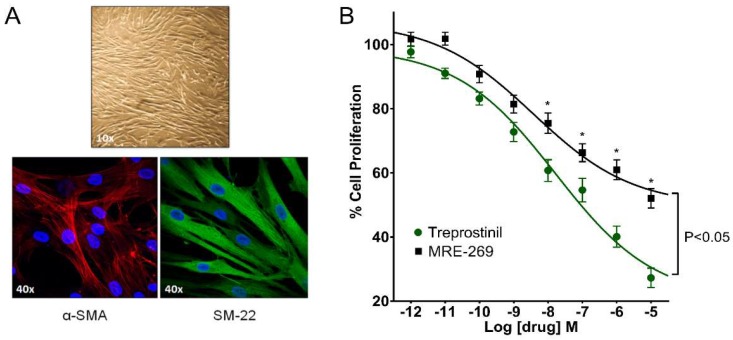
Characterization of human pulmonary arterial smooth muscle cells (HPASMCs) derived from PAH patients: comparison of the anti-proliferative effects of treprostinil and MRE-269. (**A**) Phase contrast image of HPASMCs grown to confluence and immunofluorescence staining using antibodies directed against smooth muscle markers, α-SMA (red) and SM-22 (green). In both cases, the nucleus is stained blue with 4’,6-diamidino-2-phenylindole (DAPI). (**B**) Concentration-response (0.001–10,000 nM) of treprostinil and MRE-269 on cell proliferation, assessed after 4 days of drug treatment using an MTS assay kit. Data are expressed as % cell proliferation relative to the growth response induced by 9% fetal bovine serum (FBS) and 3 nM endothelin-1 (ET-1) alone (100%). Significance was tested using two-way ANOVA with Bonferroni post-hoc correction. * *p* < 0.05 when compared to treprostinil. Data-sets were acquired using cells from the same patients (10–11 independent experiments, from 5 patient isolates; passage 3–7).

Human PASMCs derived from patients with PAH showed classic ‘hill and valley’ morphology (Figure 1A). A high percentage of cells (close to 100%) stained positive for both the smooth muscle markers, α-smooth muscle actin (α-SMA) and SM-22 (Figure 1A and Figure S2), but not the endothelial cell markers, cluster of differentiation 31 (CD-31) or von Willebrand factor (vWF; Figure S2), confirming their likely origin as smooth muscle cells. We have previously shown via Western blotting that these cultured HPASMCs also express smooth muscle myosin heavy chain and caldesmon, markers not routinely expressed in either fibroblasts or myofibroblasts [16]. However, we cannot exclude the possibility that our cell population might contain myofibroblasts, which stain for α-SMA (Figure S2).

To assess the concentration-dependent effects of putative anti-proliferative agents, HPASMCs were incubated in smooth muscle basal medium (SMBM) containing 9% fetal bovine serum (FBS) plus 3 nM ET-1 for 4 days. This combination of ET-1 and FBS was used to provide a synergistic stimulus for evoking the proliferation of HPASMCs, as described by others [22]. In cells incubated with treprostinil, a concentration-dependent reduction in proliferation (as measured by MTS assay) was observed over a wide concentration range (0.001–10,000 nM; Figure 1B). Significant (*p* < 0.05) anti-proliferative actions were seen at subnanomolar concentrations (0.1 nM) of treprostinil. The IC_50_ for treprostinil was 11 nM, with an inhibition of cell growth of 73% occurring at 10,000 nM. The non-prostanoid IP receptor agonist, MRE-269 [10], also caused a concentration-dependent reduction in HPASMC proliferation (Figure 1B). Significant (*p* < 0.05, *n* = 10) anti-proliferative actions of MRE-269 were seen at 1 nM and higher, although the degree of inhibition between 10 and 10,000 nM was significantly less than with treprostinil, being only 48% at 10,000 nM (Figure 1B). The estimated IC_50_ for this anti-proliferative action of MRE-269 was 4 nM. Thus, despite MRE-269 having a slightly higher binding affinity (Ki 20 nM) than treprostinil (Ki 32 nM) at the human IP receptor [10,13], the threshold for this drug to significantly inhibit proliferation occurred at a 10 fold higher concentration than seen with treprostinil. The greater anti-proliferative response elicited by treprostinil compared to MRE-269 may suggest that treprostinil, as well as activating the IP receptor, signals through an additional target to inhibit cell growth as previously reported in HPASMCs from PAH patients [16].

### 2.3. Role of IP Receptors

**Figure 2:**
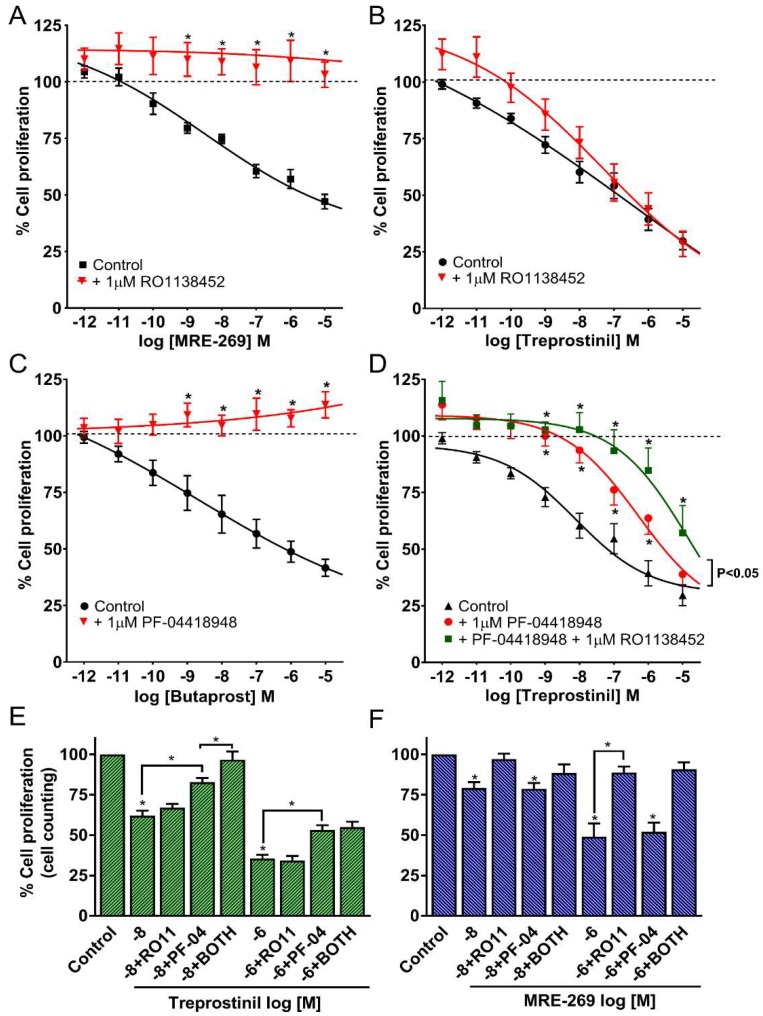
Differential role of prostanoid IP and EP_2_ receptors in mediating the anti-proliferative effects of treprostinil and MRE-269 in PAH cells. The anti-proliferative effects of MRE-269, treprostinil and butaprost in the absence and presence of the IP receptor antagonist, RO1138452 (**A,B**), the EP_2_ receptor antagonist, PF-04418948 (**C**,**D**) or in combination (**D**). Human PASMCs were left untreated (Control) or treated with 10 nM (log −8) and 1000 nM (log −6) of either treprostinil (**E**) or MRE-269 (**F**) in the absence or presence of the IP receptor antagonist, RO1138452 (RO11; 1 µM), the EP_2_ receptor antagonist, PF-04418948 (PF-04; 1 µM) or a combination (BOTH). Antagonists (1 µM) were added 30 min prior to the receptor agonists. Cell proliferation was assessed in HPASMCs from PAH patients after 4 days of drug treatment using an MTS assay kit (**A**–**D**) or by cell counting (**E**,**F**). Data expressed as % cell proliferation relative to the growth response. Significance was tested using one or two-way ANOVA with Bonferroni post-hoc correction (**A**–**D**) or Newman–Keuls multiple comparison test (**E**,**F**). * *p* < 0.05 when compared to receptor agonist alone (**A**–**D**), control (**E**,**F**) or as indicated. Each comparative data-set was acquired using cells from the same patients (4–6 isolates, passage 3–9).

To evaluate the role of IP receptors in the anti-proliferative activity of MRE-269 and treprostinil, HPASMCs were concurrently incubated with the selective IP receptor antagonist RO1138452 (CAY10441; 1 μM). This concentration of RO1138452 was previously shown to antagonize IP receptors in a number of systems [7,16,23]. The dose-dependent, anti-proliferative response at all concentrations of MRE-269 (0.001–10,000 nM) was abolished in the presence of RO1138452 (Figure 2A). A more complex pattern was observed when treprostinil was incubated concurrently with RO1138452. At very low treprostinil concentrations (≤0.1 nM), the responses were abolished by RO1138452 (1 µM), whereas above these concentrations there was some or no reduction in anti-proliferative activity (Figure 2B). Overall, RO1138452 (1 µM) did not significantly shift (*p* = 0.573 for interaction) the concentration-response curve to treprostinil, nor did it affect the value for I_Max_ (Table S2). Taken together, these data imply that prostanoid IP receptors are entirely responsible for the anti-proliferative properties of the active selexipag metabolite, MRE-269. This contrasts with treprostinil, where non-IP receptor targets appear to play a major role in the action of treprostinil in HPASMCs from PAH patients over a broad range (1–10,000 nM) of the drug. The latter observation supports our previously published data, where RO1138452 failed to inhibit the anti-proliferative effects of treprostinil in HPASMCs from PAH patients, though, in that study, only treprostinil concentrations outside the therapeutic range (100 nM or above) were assessed [16]. Likewise, the adenylate cyclase inhibitor, 2′,5′-dideoxyadenosine failed to inhibit the effects of treprostinil on cell growth in PAH cells, whereas it did in normal HPASMCs [9,16], as did the IP receptor antagonist, RO1138452 [16]. This suggests a switch in the mechanism of action of treprostinil from one that involves the IP receptor and cAMP in normal cells to one that largely does not in diseased cells.

### 2.4. Role of EP_2_ Receptors

Involvement of EP_2_ receptors in modulating growth responses in HPASMCs from PAH patients was evaluated by two approaches. Firstly, the highly selective EP_2_ receptor agonist, butaprost [24], caused a concentration-dependent (0.001–10,000 nM) reduction in cell proliferation, with significant (*p* < 0.05) effects seen at concentrations of 1 nM and higher (Figure 2C). The IC_50_ of butaprost was 5 nM (Table S2), and the degree of inhibition at 10,000 nM was 58%. Secondly, PF-04418948, a potent (IC_50_ of 16 nM against recombinant EP_2_ receptors) and highly selective (>2000-fold) antagonist at the human prostanoid EP_2_ receptor [25], abolished the anti-proliferative effects of butaprost at all concentrations evaluated (Figure 2C). These results are thus consistent with the presence of functional EP_2_ receptors in our smooth muscle cells isolated from PAH patients.

Likewise, PF-04418948 (1 µM) significantly reduced the anti-proliferative effects of treprostinil (*p* < 0.05), causing a significant rightward shift of the concentration-response curve by 2–3 orders of magnitude (Figure 2D). Thus, PF-04418948 (1 µM) not only significantly increased the IC_50_ of treprostinil by 67-fold (from 11 to 741 nM), as calculated from the data in Table S2, but 100 nM now became the lowest treprostinil concentration to cause the significant inhibition of cell proliferation. These data, therefore support the notion that EP_2_ receptors are being activated by treprostinil. It is relevant to note that plasma concentrations of treprostinil after subcutaneous or intravenous infusion in clinical studies are in the range of 2.5–25 nM [26], suggesting the EP_2_ receptor-based anti-proliferative actions of this drug are likely to be activated at therapeutic concentrations of treprostinil.

To evaluate interactions between EP_2_ and IP receptors, PF-04418948 (1 µM) was combined with RO1138452 (1 µM). With this combination of antagonists, there was a significant (*p* < 0.05, two-way ANOVA) further shift in the concentration-response curve for treprostinil (Figure 2D), with an IC_50_ of 3162 nM (Table S2). Similar qualitative results to those above were obtained when cell proliferation was assessed by cell counting. Thus, PF-04418948 (1 µM) significantly reversed the anti-proliferative effects of treprostinil at 10 and 1000 nM, but not the anti-proliferative effects of MRE-269 (Figure 2E,F). On the other hand, RO1138452 (1 µM) fully reversed MRE-269 responses and further inhibited treprostinil responses (at 10 nM but not 100 nM) when combined with PF-04418948 (Figure 2E,F).

Thus, our results suggest that both EP_2_ and IP receptors are activated by a broad range of treprostinil concentrations, and that receptor-driven, anti-proliferative effects of IP activation are more fully unmasked under conditions of marked EP_2_ antagonism. In contrast, the anti-proliferative actions of MRE-269 in HPASMCs from PAH patients appear to be driven solely by the IP receptor.

### 2.5. Knockdown of the EP_2_ Receptor with siRNAs

**Figure 3:**
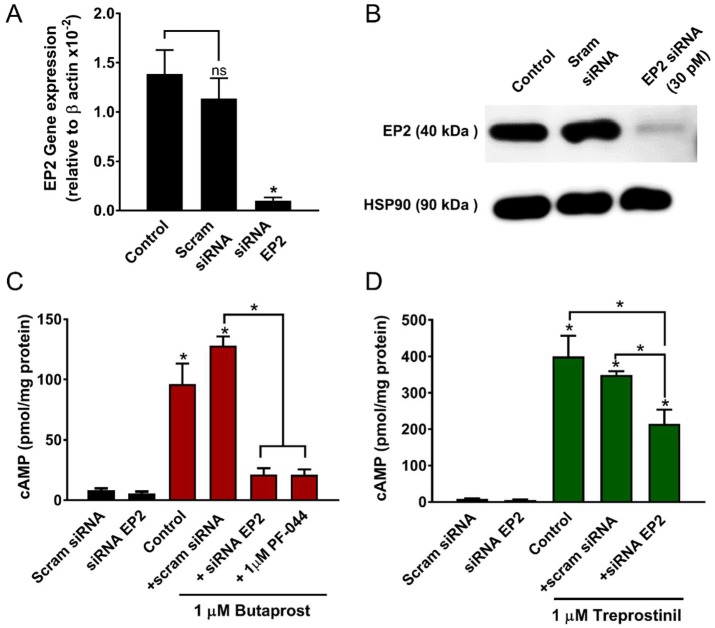
The consequence of EP_2_ gene silencing on cAMP levels in HPASMCs derived from PAH patients. Cells were starved for 48 h, transfected with 30 pM of EP_2_ receptor small interfering RNA (siRNA) or its scrambled (scram) negative control and then grown for 4 days. EP_2_ receptor expression was assessed by RT-qPCR (**A**) and by Western blotting (**B**), with levels normalised to *β-actin* or HSP90, respectively, for quantification of siRNA effects by imageJ. Intracellular cAMP was measured in growing cells after a 30 min application of butaprost (**C**) or treprostinil (**D**) in the absence (control) or presence of either scrambled (scram) siRNA, siRNA against the EP_2_ receptor or PF-04418948 (1 µM). Basal levels of cAMP were also measured in the presence of siRNA constructs without agonist. ns = non-significant. * *p* < 0.05 compared to either scram siRNA or as indicated; one-way ANOVA with Newman–Keuls multiple comparison test (*n* = 3–8 independent experiments; passage 2–10).

In HPASMCs from PAH patients, EP_2_ receptor mRNA expression was reduced by 90.9 ± 3.2% (*n* = 3) and protein levels were reduced by 76.3 ± 7.9% (*n* = 5; *p* < 0.05) following 4 days of treatment with EP_2_ receptor siRNAs (30 pM) compared with scrambled siRNA (Figure 3A,B). The lack of significant effects of the scrambled siRNA (negative control) is indicative of sequence-specific silencing under our experimental conditions rather than from the non-specific effects of RNA interference *per sey*.

Moreover, cAMP levels elevated by butaprost (1000 nM) were almost fully inhibited by EP_2_ siRNAs or 1 µM PF04418948 (*p* < 0.05; Figure 3C), consistent with it being a highly selective EP_2_ agonist in radioligand binding studies [24], and as also proposed from gene deletion studies in mice [27]. The stimulation of cAMP levels by treprostinil (1000 nM) was, however, only partially inhibited by EP_2_ receptor siRNAs (Figure 3D). It should be noted that treprostinil produced roughly 3.5 times more cAMP than a similar concentration of butaprost. The most logical explanation is that treprostinil activates both IP and EP_2_ receptors but that the former receptor is more efficiently coupled to cAMP generation in HPASMCs. Indeed, the EP_2_ siRNA reduced cAMP levels roughly by the same amount as that elevated by butaprost. This concurs with previously reported data concluding that the major stimulus for cAMP production induced by treprostinil comes via the IP receptor [16].

**Figure 4:**
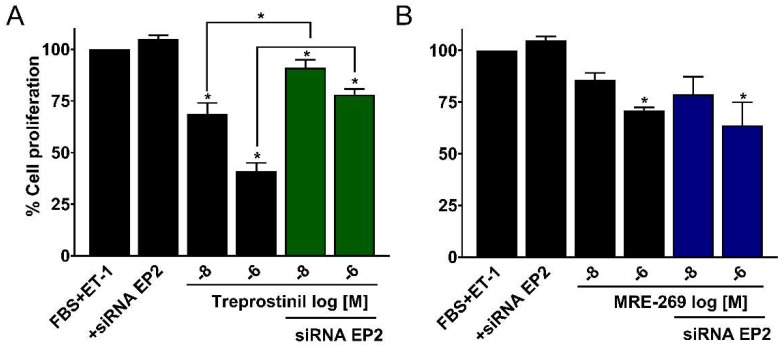
Knockdown of EP_2_ receptors with siRNAs has a differential effect on the anti-proliferative actions of treprostinil and MRE-269 as assessed by cell counting. Distal HPASMCs from PAH patients were growth-arrested for 48 hr. Cells were then grown and either left untreated (Control), or treated with 10 nM (log −8) and 1000 nM (log −6) of either treprostinil (**A**) or MRE-269 (**B**) with or without EP_2_ receptor siRNAs. After 4 days, cells were counted and the data expressed as % cell proliferation relative to growth response induced by 9% FBS and 3nM ET-1 (100%). Data are shown as mean ± S.E.M. (*n* = 4; passage number 6–9). * *p* < 0.05 when compared to control or as shown (1 way ANOVA with Newman–Keuls multiple comparison test).

In cell counting experiments, the anti-proliferative effects of 10 and 1000 nM treprostinil on HPASMCs were significantly reduced (*p* < 0.05, *n* = 4) by EP_2_ receptor siRNAs, whereas MRE-269 effects were not (Figure 4A,B). Such observations support the proliferation experiments using selective pharmacological antagonists, and again highlights the key pharmacological differences between these two prostacyclin mimetics, with different prostanoid receptors playing a major role in mediating the anti-proliferative effects of either treprostinil or MRE-269.

### 2.6. Prostanoid Receptor Expression in Cultured HPASMCs and Patient Lung Sections

**Figure 5:**
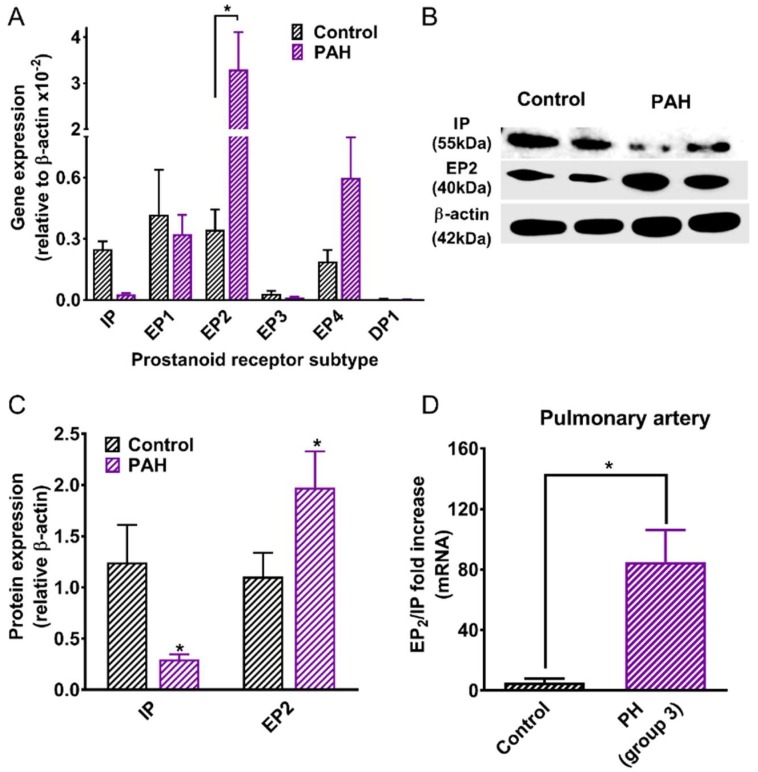
Expression of prostanoid IP and EP_2_ receptors, as determined by RT-qPCR and Western blotting in cultured HPASMCs and pulmonary artery from control and pulmonary hypertensive patients. (**A**) Messenger RNA was extracted from growing HPASMCs isolated from control and PAH patients and converted to cDNA. The data were normalised to the housekeeping gene, *β-actin* and relative gene expression determined using the 2^−^^ΔΔ*Ct*^ method. * *p* < 0.05, 2-way ANOVA with Bonferroni correction (3 samples per cell isolate from *n* = 4 patients; passage 2–9). (**B**) Protein levels of the IP receptor, the EP_2_ receptor and β-actin were visualised by Western blotting. (**C**) Band density was analysed by ImageJ and the densitometry data were normalised to the housekeeping gene, *β-actin*. * *p* < 0.05, 2-way ANOVA, with Holm–Sidak’s correction (*n* = 4–6 patient samples, passage 5–9). (**D**) Relative EP_2_ to IP receptor mRNA expression in human pulmonary arteries isolated from controls and group 3 pulmonary hypertensive patients. * *p* < 0.05, unpaired *t*-test (*n* = 4 patient arteries).

Real-time quantitative PCR (RT-qPCR) was used to determine the relative expression of prostanoid receptor mRNA in growing (non-synchronised) HPASMCs derived from normal and PAH patients (Figure 5A). In normal cells, IP, EP_1_, EP_2_, and EP_4_ receptors were moderately expressed and to a similar level. In PAH cells, the ratio of EP_2_/IP expression increased from 1.4 (normal) to 115 (*p* < 0.05, unpaired *t*-test) due to a fall in IP receptor expression and a concomitant rise in EP_2_ levels (*p* < 0.05). EP_4_ levels also rose 6-fold in the PAH cells, while EP_1_ levels remained unchanged, though the expression of both was significantly less (*p* < 0.05, one way ANOVA) compared to EP_2_, being 5- and 11-fold lower, respectively. EP_3_ receptors were moderately to weakly expressed, while DP_1_ receptors were very weakly expressed, often not detectable in samples. In Western blotting experiments, IP receptor protein levels were reduced while EP_2_ receptor protein levels were higher (*p* < 0.05; when comparing normal and PAH samples, Figure 5B,C). Furthermore, in pulmonary arteries isolated from patients with group 3 pulmonary hypertension (PH), the ratio of EP_2_/IP receptor expression was elevated (*p* < 0.05) compared to the controls (Figure 5D).

**Figure 6:**
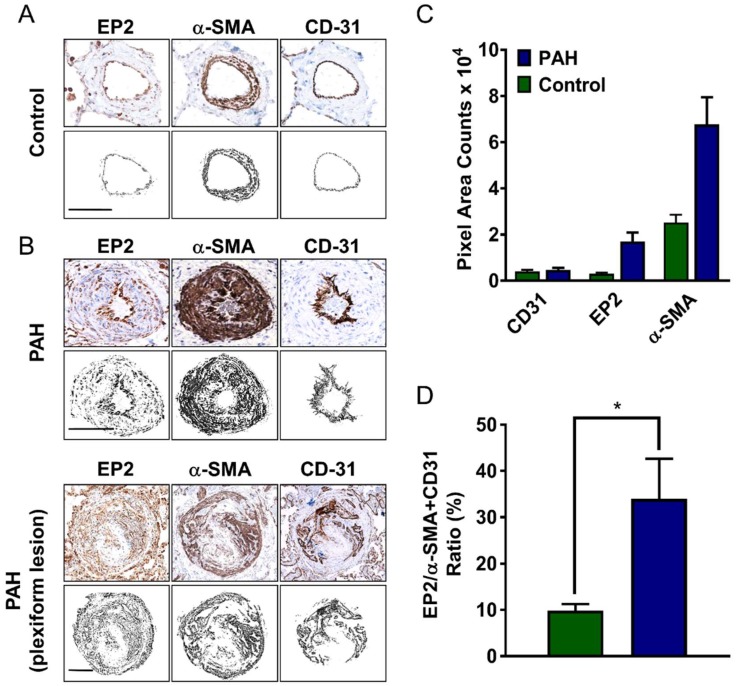
EP_2_ receptor expression increases in distal human pulmonary arteries from PAH patients. Representative immunohistochemical staining in serial sections of a pulmonary artery from a control (**A**) and a PAH patient (**B**) showing the prostanoid EP_2_ receptor (EP_2_), the smooth muscle marker, the α-smooth muscle actin (α-SMA) and the endothelial cell marker, CD-31. Staining was visualised by diaminobenzidine (brown) in sections counterstained with haematoxylin (blue) and quantified using ImageJ. Grey images represent the digitisation of staining in arterial sections, where adventitial staining has been excluded to focus on the expression in muscle and the endothelium. Bars are 100 µm and is the same for each section. The data are expressed as the pixel area of the respective staining for CD-31, α-SMA, and EP_2_ receptor (**C**) or as the ratio of EP_2_/α-SMA+CD31 area staining (**D**). Excluded from the analysis of data presented in parts (**C**) and (**D**) was staining in plexiform lesions. * *p* < 0.05 unpaired *t*-test (*n* = 20–23 sections from 7 controls and 8 PAH patient samples).

The EP_2_ receptor expression was examined in lung arterial sections from control and PAH patients (Figure 6). EP_2_ receptors were visibly expressed in the endothelium of normal distal (small muscular terminal bronchiolar and intra-acinar) arteries as observed by co-localisation of the endothelial marker, CD-31, whereas in the medial layer staining was more discrete and punctate (Figure 6A). EP_2_ receptor staining was also apparent in cells contained within the adventitia, where α-smooth muscle actin (α-SMA) staining was absent (Figure 6A). In the PAH sections, EP_2_ receptor staining was increased in the medial and adventitial layer (left panels) and appeared stronger in and around the plexiform lesion (Figure 6B). The quantification of staining in small arteries (excluding plexiform lesions) showed differences between normal and PAH sections, with increases in EP_2_ and α-SMA pixel area counts but with little or no change in CD-31 observed in PAH sections (Figure 6C). The area of EP_2_ staining coinciding with α-SMA and CD-31 staining (shown as a ratio) was significantly increased (*p* < 0.05) in PAH arteries (Figure 6D). In proximal (larger muscular pre-acinar) pulmonary arteries, EP_2_ staining was weak in the normal artery and largely confined to the medial layer (Figure S3A). In PAH tissue, EP_2_ receptor staining was substantially increased in the adventitial layer, with some increased staining in the medial layer (Figure S3B), though in contrast to small arteries, this was not significantly increased relative to α-SMA staining (Figure S3C). We conclude that the EP_2_ receptor is robustly expressed in pulmonary artery cells and tissue from patients with either group 1 or group 3 pulmonary hypertension, contrasting with the IP receptor, which is downregulated, as previously reported in PAH [16,28]. Likewise, in experimental PAH, downregulation of the IP receptor in distal arteries has been reported while EP_2_ receptor expression remained unchanged [28], suggesting that pulmonary disease itself may negatively impact on the IP receptor expression. 

## 3. Discussion

The current studies have now identified a key role of prostanoid EP_2_ receptors in the regulation of human pulmonary arterial smooth muscle proliferation. Following the identification of treprostinil as a potent activator of prostanoid EP_2_ receptors [13], we now demonstrate for the first time using the EP_2_ receptor antagonist, PF-04418948 [25], as well as EP_2_ receptor siRNAs, that the anti-proliferative effect of treprostinil at therapeutic doses appears largely dependent on activation of the EP_2_ receptor in HPASMCs from PAH patients. We also now demonstrate that the non-prostanoid IP receptor agonist MRE-269 has anti-proliferative activity, though unlike treprostinil, its activity is abolished by the highly selective IP receptor antagonist, RO1138452 [16] and is not affected by either PF-04418948 or EP_2_ siRNAs. This implies a predominant or sole role for the IP receptor in the anti-proliferative actions of MRE-269 in HPASMCs from PAH patients, and by extrapolation, of its parent molecule selexipag.

The earlier findings using RO1138452 to antagonise IP receptors [16] have now also been extended to explore a more extensive range of treprostinil concentrations (1 pM to 10 µM). This new work in HPASMCs from PAH patients demonstrates that when EP_2_ receptors are inhibited with PF-04418948, RO1138452 causes a significant rightward shift of the concentration-response curve to treprostinil. This suggests both EP_2_ and IP are activated by a broad range of treprostinil concentrations and that the receptor-driven anti-proliferative effects of IP activation are more fully unmasked under conditions of substantial EP_2_ antagonism. However, RO1138452 generally failed to substantially antagonise responses at higher concentrations of treprostinil (10 nM or greater), further suggesting that non-IP receptor targets contribute. This contrasts with studies in normal human PASMCs and in normal human proximal pulmonary arteries, where the anti-proliferative and vasorelaxation responses to treprostinil were abolished by RO1138452, consistent with a major role of the IP receptor [7,16]. The extent to which EP_2_ receptors are functionally active in normal distal pulmonary arteries and cultured PASMCs is unknown and should in the future be investigated.

To confirm the presence of functional EP_2_ receptors in our PAH cells, we used butaprost, a selective EP_2_ receptor agonist, which has little to no activity at the human IP receptor (Ki ~ 100 µM) or any other prostanoid receptor [24] and fails to elicit an anti-proliferative response in EP_2_ receptor null-mice [20], or as in this study, to significantly elevate cAMP after treatment with EP_2_ siRNAs. The threshold to significantly inhibit proliferation was 1 nM, similar to that previously reported for the inhibition of proliferation in murine aortic cells [20]. Consistent with the specificity of butaprost, PF-04418948 abolished its anti-proliferative effects over the entire concentration range and inhibited the anti-proliferative activity of treprostinil, substantially shifting the concentration-response curve over the entire range. Likewise, EP_2_ receptor siRNAs substantially reversed the treprostinil effects on cell growth, clearly demonstrating a predominant contribution of EP_2_ receptors. The activation of EP_2_ receptors, in addition to the IP receptor, may in part account for the higher maximal anti-proliferative response to treprostinil compared to that seen with the IP-selective agonist, MRE-269. Of note, the EP_2_ receptor does not undergo rapid agonist-induced desensitization in vitro [29], whereas the IP receptor does [5,29]. This suggests signalling via EP_2_ receptors may give rise to longer lasting beneficial effects in PAH, and may provide another option in those patients seen not to be responding well to selexipag and subsequently identified with low IP expression or limited functional capacity of this receptor. It should be noted that the anti-proliferative responses to treprostinil at higher concentrations (1 µM and above) were not fully inhibited in the presence of both IP and EP_2_ receptor antagonists suggesting an additional mechanism may be operating at the higher doses. This could involve the peroxisome proliferator-activated receptor-γ (PPARγ), which via a mechanism that appears independent of cAMP generation, could play a significant role in mediating the anti-proliferative effects of treprostinil in human PASMCs isolated from PAH patients [16].

We observed a striking difference in the pattern of prostanoid receptor mRNA expression in HPASMCs derived from control versus PAH patients. While prostanoid receptor mRNA was similarly expressed in control cells, with the exception of EP_3_ and DP_1_ which were much lower, the relative expression of EP_2_ over the IP receptor was enhanced 84-fold at the message level and 7-fold at the protein level in PAH cells. At this stage, it is impossible to gauge if the medication given to PAH patients influenced our current findings in HPASMCs and pulmonary arteries obtained from these patients. Nonetheless, enhanced gene expression of EP_2_ receptors compared to controls has been reported in airway smooth muscle cells [17] and also in lung fibroblasts [30] derived from patients with asthma or chronic obstructive pulmonary disease, respectively. Furthermore, enhanced EP_2_ receptor expression was noted during neointimal proliferation and was reported to underlie the increased anti-proliferative effects of PGE_2_ and butaprost treatment in airway smooth muscle cells from asthmatics, and to be up-regulated in response to platelet-derived growth factor [17,20] and transforming growth factor β [19], key drivers of smooth muscle proliferation in PAH [18]. The opposite relationship was observed for IP receptor expression, which was markedly reduced in PAH HPASMCs, supporting previous observations that this receptor is down-regulated either as a consequence of disease or the PAH therapy [16,28]. Likewise, we found in pulmonary arteries from PH patients, that the rise in EP_2_ to IP receptor mRNA expression ratio compared to controls could largely be accounted for by a fall in IP receptor expression (not shown). Similarly, in a rat monocrotaline model of PAH, mRNA levels for IP, EP_1_ and EP_3_ were all down-regulated in distal PASMCs, whereas the EP_2_ and EP_4_ receptor expression was essentially unaltered [28]. Irrespective of IP receptor downregulation, treprostinil reversed monocrotaline-induced vascular medial thickening in the rat [31]. Taken together, EP_2_ receptors appear to be more robustly expressed in human pulmonary tissue in PAH compared to IP receptors, which appear more labile.

Previous reports suggested that in large human pulmonary artery vessels, EP_2_ receptors are weakly functional because of an active EP_3_ system [32], though curiously high sensitivity to EP_2_ agonists was noted in some instances [33]. We observed a far stronger staining of EP_2_ receptors in small versus large arteries, suggesting EP_2_ receptors may play a greater role in small pulmonary vessels. Although the functional consequence of activating these receptors in the lung requires investigation, studies in EP_2_^−/−^ gene-deleted mice show that EP_2_ receptors regulate blood pressure and underpin the vasodilator response to PGE_2_ [27].

This high expression of EP_2_ receptors in HPASMCs and small blood vessels from the lungs of patients with end-stage PAH contrasts with the weak staining for the IP receptor and PPARγ previously reported in the intimal proliferating cells of distal arteries from IPAH patients [16]. The role of EP_2_ receptors in the context of remodelling in PAH is unknown, though neointimal hyperplasia in response to femoral artery injuries was markedly accelerated in EP_2_^−/−^ mice and associated with the increased proliferation and migration of vascular smooth muscle cells [20] and fibroblasts [34], suggestive of a protective role of EP_2_ receptors in vascular remodelling.

EP_2_ receptor staining was observed in the adventitial layer of arteries and in plexiform lesions in lung sections from PAH patients. The adventitial staining is likely to come from fibroblasts, which reside predominately in this layer, undergoing proliferation and producing significant amounts of collagen to increase adventitial thickness [35,36]. EP_2_ receptor staining may also come from inflammatory cells, particularly monocytes and dendritic cells, which also reside in the adventitia of remodelled arteries in PAH [18,35]. Thus, the elevated EP_2_ receptor expression relative to other prostanoid receptors found in the current study may reflect its up-regulation as a consequence of the disease. Importantly, EP_2_ receptors have a range of inhibitory actions on fibroblast function that could be beneficial in PAH [34,37].

The current study provides strong evidence for a key role of prostanoid EP_2_ receptors in the anti-proliferative effects of treprostinil on PASMCs from PAH patients. This contrasts with prostanoid IP receptors that appear to be entirely responsible for the anti-proliferative properties of MRE-269, the active metabolite of selexipag. The broader pharmacological receptor profile of treprostinil may be important in pathologic conditions such as in PAH where down-regulation of the IP receptor occurs. Indeed, this current data strongly suggest that the activation of the more robust and highly expressed EP_2_ prostanoid receptor pathway, in concert with or in lieu of IP receptor signalling, makes an important contribution to the therapeutic activity of treprostinil. Thus, the EP_2_ receptor represents a previously unrecognised modulator of human pulmonary vascular cell proliferation, and hence remodelling, which has clinical implications for the treatment of PAH.

## 4. Materials and Methods

### 4.1. Source, Isolation, and Culture of PASMCs from Hypertensive and Normal Patients

Lung tissue was taken after patient consent or the consent of a relative and with the Ethics Committee approval from the Great Ormond Street Hospital (ICH and GOSH REC 05/Q0508/45, 11/4/05 and 16/3/2010) and the Assistance Public–Hôpitaux de Paris (IRB00006477, agreement No. 11-045, 31/3/11). Samples were obtained from patients (*n* = 10) diagnosed as having IPAH who went on to have a transplant after failed treatment or who had died. Tissues were also obtained from patients with pulmonary hypertension due to lung diseases and/or hypoxia (group 3 classification) where the mean pulmonary artery pressure (mPAP) was 30 ± 3 mmHg. For controls, donor lungs not suitable for transplantation, but otherwise histologically normal, or parenchymal strips from macroscopically normal regions of lungs from patients with suspected malignancy, were used (*n* = 8).

Primary cell lines of distal HPASMCs were derived by enzymatic dissociation as previously described [16] and grown at 37 °C in a humidified atmosphere of 5% CO_2_ in human smooth muscle basal medium (SMBM; Lonza, Slough, UK) containing 9% FBS (Life Technologies, Paisley, UK) and penicillin/streptomycin (45 units/mL Life Technologies). After reaching confluence, cells were washed with phosphate-buffered saline (PBS; Life Technologies) and treated with 0.25% trypsin-EDTA (Life Technologies) for further passage. Only cells between passage 2 and 10 were used in experiments.

### 4.2. Cell Proliferation Assays

To assess the concentration-dependent effects of putative anti-proliferative agents (0.01–10,000 nM), HPASMCs from PAH patients were seeded onto 96- (MTS assay) or 6- (cell counting) well plates at a density of 1 × 10^4^ cells/mL (total volume 100 μL or 2 mL, respectively). Cells were grown in SMBM containing 9% FBS, and after 24 h, the media was replaced with just SMBM for 48 h to growth-arrest cells. Subsequently, the cells were incubated in SMBM containing 9% FBS plus 3 nM ET-1 for 4 days in the absence and presence of 0.1% dimethyl sulphoxide (DMSO), with and without the test agent. Proliferation responses were compared to cells incubated with no added growth factors over the same time period (the time control). Each intervention was performed in quintuplicate (MTS) or in duplicate (cell counting).

In the majority of experiments, proliferation was assessed using an MTS cell proliferation assay kit (Promega, Southampton, UK), a colorimetric method for determining the number of viable cells based on the cleavage of MTS (3-(4,5-dimethylthiazol-2-yl)-5-(3-carboxymethoxyphenyl)-2-(4-sulfophenyl)-2H-tetrazolium, inner salt) to formazan by cellular mitochondrial dehydrogenases. An increase in cell number leads to a proportional increase in the amount of formazan dye formed, which can be quantified by measuring the absorbance of the dye solution at 490 nm using a Versamax Microplate Reader (Sunnyvale, CA, USA). For each drug concentration, the absorbance was measured from five wells and the average was taken. The background absorbance was corrected by subtracting the average absorbance from the ‘no cell’ control wells from all other absorbance values. In other experiments (Figure 2E,F and Figure 4), cell number was counted using an automated cell counter (ADAM; Digital Bio, Seoul, Korea), which provides counts of the total and non-viable cells using the fluorescent DNA binding dye, propidium iodide in lysed and non-lysed cells, respectively. Cell proliferation was normalized to the growth response without the solvent (taken as 100%) and shown as the % cell proliferation. Comparison of the agonist effects were made in the same patient cell isolates, usually at a similar passage number with experiments run in parallel under identical conditions and proliferation assays performed on the same day.

### 4.3. Transfection of Small-Interfering RNA (siRNAs) Against EP_2_ Receptors

Human PASMCs from PAH patients were seeded onto 6-well plates, and after 24 h, they were growth arrested in serum-free SMBM (Lonza, UK) for 48 h. Cells were then transfected according to the manufacturer’s instructions. Briefly, the siRNA (ON-TARGETplus SMARTpool *PTGER2*; Dharmacon, Cambridge, UK) was diluted in Dharmafect while lipofectamine (Invitrogen, Paisley, UK) was made up in an OptiMen-1 buffer (Invitrogen). The two were then mixed in a 1:1 ratio and left for 20 min at room temperature. Cells were transfected in the growth medium containing penicillin/streptomycin (Life Technologies) in the absence or presence of 30 pM of EP_2_ receptor siRNA or the scrambled negative control (Dharmacon, UK), added 4 h prior to the addition of agonists. After 4 days, the cells were processed for Western Blotting, cAMP measurements, and qPCR as described below or the cells were counted in proliferation assays as described above.

### 4.4. Western Blotting

Cells were lysed in RIPA buffer (Sigma-Aldrich, Gillingham, Dorset, UK) containing phosphatase inhibitors, and centrifuged at 900× *g* for 15 min at 4 °C; the resulting supernatant was stored at −80 °C until use. Protein samples (10 µg) were run on a NuPAGE^@^ Bis-Tris gel (Invitrogen, Paisley, UK) alongside pre-stained molecular weight markers (Fermantas, Cambridge, UK) and then transferred electrophoretically to PVDF membranes (Invitrogen). Blots were washed in PBS containing 5% skimmed milk and 0.1% Tween-20 (PBST) before being incubated overnight at 4 °C with primary antibodies diluted in PBST against EP_2_ receptor (1:1000 Cayman Cat No. 101750; Cambridge Bioscience, Cambridge, UK) and HSP90 (1:1000; Cell Signaling Technology, Cat No. 4877; Hitchin, UK) and then the appropriate secondary antibody for one hour at room temperature. To ensure equal amounts of protein loading, the blots were stripped (RE-BLOT PLUS Western Blot Stripping Solution, Cat. No. 2502, Merk Millipore, Watford, UK) and re-probed with an anti-β-actin antibody. Protein bands were visualized using the enhanced chemiluminescence plus reagent detection system (GE Healthcare, Little Chalfont, Buckinghamshire, UK) and imaged via a Gel-Doc system (Snygene; Cambridge, UK). ImageJ (National Institute of Mental Health, Bethesda, MD, USA) was used to compare the density of the bands relative to β-actin for both the IP and EP_2_ prostanoid receptor protein.

### 4.5. Cyclic AMP Extraction and Measurement

Human PASMCs were incubated with either butaprost or treprostinil for 30 min and the reaction was stopped by aspirating the media and washing cells with 1 mL of cold PBS. Cyclic AMP was extracted from cells by lysing them in 0.1 M HCl for 20 min on ice, followed by centrifugation of the suspension at 1000× *g* for 10 min at 4 °C. Intracellular cAMP was measured using a competitive enzyme immunoassay according to the manufacturer’s instructions (ADI-900-163; Enzo Life Sciences, Exeter, UK). The protein concentration in the supernatant was determined using a bicinchoninic acid (BCA) protein assay kit (Novagen, Watford, UK) and cAMP normalised per mg of protein.

### 4.6. Real-Time Quantitative PCR (RT-qPCR)

#### 4.6.1. Cultured HPASMCs

Quantitative PCR (qPCR) was used to determine the relative expression of different prostanoid receptors using a broadly similar method to that previously published [38]. Cultured HPASMCs were lysed and treated with TRIzol reagent (Life Technologies, UK) which was mixed with chloroform, centrifuged, and the aqueous phase then combined with propran-2-ol. Following this, the sample was incubated at −20 °C for 1.5 h and the total RNA pellet isolated by centrifugation. The pellet was then washed twice in 75% ethanol and dissolved in 25 μL of nuclease-free water (Life Technologies, UK). The concentration and purity of RNA was determined using a NanoDrop-1000 Spectrophotometer (Thermo Scientific, Wilmington, DE, USA) by measuring the optical density between 260 and 280 nM (260/280) and between 260 and 230 nM (260/230). Only samples with ratio values of 260/280 and 260/230 within the range 1.7–2.0 were accepted as good quality RNA.

Complementary DNA (cDNA) was synthesised from 500 ng of total RNA in a reverse transcription reaction mixture containing MultiScribe Reverse Transcriptase (1.25 Unit/μL), dNTP (ATP, CTP, GTP, UTP; 500 μL each), 2.5 μM Oligo(dT)16 (to ensure the transcription of mRNA but not ribosomal or transfer RNA), RNase inhibitor (0.4 Unit/μL), MgCl_2_ (5.5 mM) and reaction RT buffer (Taqman Reverse Transcription Reagents kit, Applied Biosystems Roche, Branchburg, NJ, USA). The sample was incubated in a thermal cycler (Techne Genius; Stone, Staffordshire, UK) for 60 min at 42 °C, 15 min at 72 °C followed by holding at 4 °C. The cDNA was stored at −20 °C until used.

The primer set of human *PGTIR* (NM_000960; IP receptor), *PTGER1* (NM_000955; EP_1_ receptor), *PTGER2* (NM_000956; EP_2_ receptor), *PTGER3* (NM_000957; EP_3_ receptor), *PTGER4* (NM_000958; EP_4_ receptor), *PGTDR* (NM_000953; DP_1_ receptor) and the reference gene *β-actin* (NM_001101) were purchased from Qiagen (Manchester, UK). Real-time qPCR (RT-qPCR) were set up in triplicate in a 284-well microtitre plate using 5 μL per well from a 25 μL mixture containing 12.5 μL of the SYBR-green solution (Applied Biosystems, Loughborough, UK), 2.5 μL of primer and 10 μL of cDNA (25 ng). RT-qPCR was performed using an automated thermal cycler (ABI Prism 7900HT Sequence Detection System; Applied Biosystems, Foster City, CA, USA). The PCR cycle was 50 °C for 2 min, 95 °C for 15 min, followed by 40 cycles at 94 °C for 15 s, 56 °C for 30 s and 76 °C for 30 s. The relative amount of cDNA was calculated using the “2^−ΔΔ*C*t^ threshold cycle” method, which involves comparing the CT values of the samples of interest with a reference gene, *β-actin*, where CT is defined as the number of cycles required for the fluorescent signal to exceed background levels [39].

#### 4.6.2. Pulmonary Artery

Arteries (3–6 mm internal diameter) were ground in liquid nitrogen and RNA was isolated using a tissue RNA kit (OMEGA bio-tek, Norcross, GA, USA). cDNA was synthesised using the Moloney murine leukaemia virus reverse transcriptase (Invitrogen, Carlsbad, CA, USA). The reaction was conducted for 90 min at 37 °C using 0.16 μg of RNA in 10 μL of the reaction mixture, 0.5 mM of M-MLV, and 0.5 µg/µL of Poly-d(T). RT-qPCR was performed using a LightCycler 480 Roche qPCR (Roche Diagnostics, Meylan, France). RT-qPCR was conducted in duplicate, with 4 µL of the cDNAs transferred to each real-time reaction together with 500 nM of primers and the SYBR Green Master Mix (Roche Diagnostics). The human PCR primer sequences were 5′-CACGAGGAGCAAAGCAAGTG-3′ (sense), 5′-AGGTCTGGGCTCTCCAGTCTT-3′ (antisense), and 5′-TGCTCCTTGCCTTTCACGA-3′ for the IP receptor; 5′-TGCTCCTTGCCTTTCACGA-3′ (sense) and 5′-TCAGAACAGGAGGCCTAAGGA-3′ (antisense) for the EP_2_ receptor; and 5′-GGGCACCCTGGGCTAAACTGA-3′ (sense) and 5′-TGCTCTTGCTGGGGCTGGT-3′ (antisense) for the *GAPDH* gene. The PCR thermal cycling conditions were preincubation at 95 °C for 5 min, followed by 40 cycles at 95 °C for 10 s, 60 °C for 30 s and 72 °C for 15 s. The relative amount of cDNA was calculated using the “2^−ΔΔ*C*t^ threshold cycle” method as described above (4.6.1.) using a different reference gene, *GAPDH*.

### 4.7. Immunofluorescent Staining

Human PASMCs or human umbilical vein endothelial cells (HUVECs; Cellworks, Buckingham, UK) were seeded into 8-chambered slides (BD Falcon, Oxford, UK) and grown in DMEM/F-12 or RPMI 1640 (Life Technologies, UK) containing serum. After reaching the required confluency, the cells were fixed with 4% paraformaldehyde (PFA; Sigma-Aldrich), followed by permeabilization in 0.1% Triton X-100 (Sigma-Aldrich) for 10 min. Aspirated cells were then washed three times with PBS, followed by a 10 min incubation at room temperature with 3% bovine serum albumin (BSA) in 0.01% Triton X-100. Both primary and secondary antibodies were diluted in 3% BSA in 0.01% Triton X-100. The primary was added to chambers and left overnight at 4 °C and the appropriate secondary antibody added for one hour at room temperature followed by the addition of the fluorescent nuclear stain, DAPI (Vector Laboratories, Southgate UK). The following primary antibodies were used: mouse monoclonal anti-α-SMA (1:1000, A-2547; Sigma-Aldrich), rabbit polyclonal anti-SM-22 alpha (1:500, ab14106; Abcam, Cambridge, UK), polyclonal rabbit anti-human vWF (1:400, A0082; Agilent Technologies, Stockport, Cheshire, UK), and mouse monoclonal anti-human CD-31 (1:400, 35285S; Cell Signaling Technology, Hitchin, UK). Alexafluor-555 goat anti-mouse IgG (1:1000, A11001; Invitrogen, Paisley, UK) was used as a secondary for α-SMA and CD-31 staining and Alexafluor-488 donkey anti-rabbit IgG (1:1000, A21206; Invitrogen, Paisley, UK) was used for SM-22 and vWF staining. Omission of the primary antibody served as a negative control. Confocal imaging was performed using a LEICA TCS SPE upright microscope (Leica Microsystems, Milton Keynes, UK) and Z-stack images were acquired and analysed using proprietary LEICA LAS X Software (Leica Microsystems).

### 4.8. Histology and Immunohistochemistry

Blocks of lung tissue from control and PAH patients were fixed and 10 µM serial sections were cut for histological examination. Two slides of each section were stained to look for gross pathological changes using either hematoxylin and eosin (H&E) staining, where nuclei stain blue/purple while cytoplasm and muscle stain a purplish red or Van Gieson (EVG) staining, which stains collagen in red, elastic fibres and nuclei in black and other tissue elements in yellow. Antibodies to α-SMA (Sigma-Aldrich, Poole, UK; Cat No. A2547), the endothelium marker, CD-31 (Abcam, Cambridge, UK; ab28364) and the EP_2_ receptor (Abcam, Cat. No. ab117270), were used to probe for their expression in proximal and distal blood vessels. Sections were incubated overnight at 4 °C with the primary antibody (diluted in PBS with 0.1% BSA at 1:300–500) followed by incubation with a biotin-conjugated secondary antibody (Abcam) for 1 h at room temperature. Sections were then developed utilizing avidin-conjugated horseradish peroxidase (HRP) and staining was visualised with diaminobenzidine (Sigma-Aldrich) in sections lightly counterstained with haematoxylin (Sigma-Aldrich). Control and PAH sections were handled in the same way, being developed on the same day and exposed to the chromagen for exactly the same length of time. Omission of the primary antibody served as a negative control. Specificity of staining was controlled with an inappropriate secondary antibody. Colour images were acquired and the results were stored digitally after examination by virtual microscopy (Hamamatsu Photonics, Welwyn Garden City, UK).

For histological analysis, the endothelial and smooth muscle layers were identified by CD-31 and α-SMA staining, respectively, in serial sections of distal pulmonary arteries. The staining area was quantified using the ImageJ colour threshold function, which filters out unwanted colours and then transforms the image into an 8-bit format. The staining area was quantified using the “Analyse Particles” function which assigns a pixel value based on the intensity of the brown staining. Staining in the adventitial layer and plexiform lesions was excluded from the quantification analysis. Data were expressed as the pixel area of the respective staining for CD-31, α-SMA or EP_2_ receptor or as the ratio of EP_2_/α-SMA+CD31 area staining.

### 4.9. Materials

MRE-269 ([4[(5,6-diphenylpyrazinyl)(1methylethyl) amino]butoxy]acetic acid was purchased from Cayman Chemical Company (Ann Arbor, MI, USA) and PF-04418948 (selective EP_2_ antagonist) was purchased from Tocris Bioscience (Bristol, UK). RO-1138452 (IP selective antagonist) and butaprost (15-deoxy-16*S*-hydroxy-17-cyclobutyl PGE_1_ methyl ester), a selective EP_2_ agonist, was purchased from Cambridge Bioscience UK and endothelin-1 peptide from Enzo Life Science (Exeter, UK). Treprostinil was supplied by the United Therapeutics Corp (Research Triangle Park, NC, USA). Stocks of all drugs were made up in sterile DSMO (Sigma-Aldrich) to a final concentration of 10 mM. Drugs were serially diluted in growth medium, with the solvent concentration in each well remaining constant (0.1%).

### 4.10. Statistical Analysis

Data are expressed as mean ± S.E.M. of n experiments from a minimum of 4 cell isolates derived from different patients. The maximal % inhibition (I_Max_) and the log concentration causing 50% inhibition (IC_50_) of cell proliferation was extrapolated from each single experiment using the variable slope sigmoidal-curve fitting routine obtained using the Prism 7 software (GraphPad, San Diego, CA, USA). The data are reported as IC_50_ (nM) values for clarity in the text or negative log (pIC_50_) values to allow appropriate pharmacological statistical evaluation in Table S2. Significance was assessed between two groups using a Student *t*-test and between multiple groups using either one-way analysis of variance (ANOVA) where Dunnett’s was used for comparisons against a control and Newman-Keuls test for multiple comparisons of different groups or by two-way ANOVA (with Bonferroni or Holm–Sidak’s multiple comparisons test) as indicated in the legend. *p*-value < 0.05 was considered significant, but only shown at the 95% confidence limit.

### 4.11. Key Principles of the Study Methodology

This work was conducted with due attention to detailed proposals recently discussed by Bonnet and colleagues [40] and Provencher and colleagues [41] concerning the limitations of the potential translation of basic research using human tissue to PAH disease presenting in patients. Thus, due care was taken regarding the isolation and purity of the HPASMCs, their histological assessment in situ and the appropriate selection of patients and cells for both control samples and PAH samples. To this end, the same protocol for cell incubation and data acquisition was used for both “control cells” and “PAH cells” along with the replication of results in multiple cell lines over a wide patient age range. In all figure legends, the number of independent biological data points and patient samples has been included. The number of technical replicates is defined in each methods section. The concept that EP_2_ receptors will be targeted (activated) at therapeutic concentrations of treprostinil has been independently verified in two further species: mouse and rabbit [14]. All datasets on which the conclusions of this article rely will be made available on request, as long as they are within ethical consideration to prevent amongst other things, patient identification.

## Supplementary Materials

Supplementary materials can be found at http://www.mdpi.com/1422-0067/19/8/2372/s1.

## Abbreviations

α-SMA	α-smooth muscle actin
BCA	bicinchoninic acid
BSA	bovine serum albumin
butaprost	15-deoxy-16S-hydroxy-17-cyclobutyl PGE1 methyl ester
CD-31	cluster of differentiation 31
DAPI	4’,6-diamidino-2-phenylindole
DMSO	dimethyl sulphoxide
ET-1	endothelin-1
EVG	Van Gieson
H&E	hematoxylin and eosin
HUVECs	human umbilical vein endothelial cells
HPASMCs	human pulmonary arterial smooth muscle cells
IC_50_	log of concentration causing 50% inhibition
I_Max_	maximal % inhibition
IPAH	idiopathic pulmonary arterial hypertension
mPAP	mean pulmonary artery pressure
MRE-269	4[(5,6-diphenylpyrazinyl)(1methylethyl) amino]butoxy]acetic acid
MTS	3-(4,5-dimethylthiazol-2-yl)-5-(3-carboxymethoxyphenyl)-2-(4-sulfophenyl)-2H-tetrazolium, inner salt
PAH	pulmonary arterial hypertension
PASMCs	pulmonary arterial smooth muscle cells
PBS	phosphate-buffered saline
PBST	phosphate-buffered saline Tween-20
PFA	paraformaldehyde
pIC_50_	negative log of concentration causing 50% inhibition
PVRI	pulmonary vascular resistance index
qPCR	quantitative PCR
RT-qPCR	Real-time qPCR
siRNAs	small interfering RNAs
SMBM	smooth muscle basal medium
vWF	von Willebrand Factor
