# Supplementary material for: Prostanoid EP2 Receptors Are Up-Regulated in Human Pulmonary Arterial Hypertension: A Key Anti-Proliferative Target for Treprostinil in Smooth Muscle Cells
Source: Int J Mol Sci. 2018 Aug 12;19(8):2372. doi: 10.3390/ijms19082372 (PMC6121445; doi:10.3390/ijms19082372)
Supplement: Supplementary file 1 [file ijms-19-02372-s001.pdf]

Table S1. Clinical Classification and characteristics of patients with pulmonary arterial hypertension (PAH)

Sex	Age (yr)	Patient Diagnosis (Nice classification) /other Info	mPAP (mm Hg)	PVRI (Wood units.m^2)	Treatment duration (yr)		
					Prostacyclin	ERA	PDE5i
**Male**	3.1	PAH associated with small defects (group 1.4.4.3)/ small ASD	69	-	Epo 0.9	Bos 0.5	Sild 0.9
**Female**	5.0	PAH associated with small defects (group 1.4.4.3)/ small ASD	73	33	Epo 2	Bos 2.3	no
**Male**	5.3	PAH associated with small defects (group 1.4.4.3)/ small ASD	89	38.9	Epo 4.2	Bos 2.5	no
**Female**	6.5	PAH associated with small defects (group 1.4.4.3)/ small ASD Kabuki Syndrome	94	53	Epo 0.5	Bos 3.2	Sild 3.3
**Male**	8.2	PAH associated with small defects (group 1.4.4.3)/ small VSD	66	18.6	Epo 3.25	Bos 1	no
**Female**	12.8	IPAH	56	35	Epo 7.5	Bos 7.6	Sild 7.6
**Female**	14.1	PAH associated with CHD after defect closure (group 1.4.4.4)/ transposition of great arteries with neonatal repair	79	23.8	Epo 1.9	Bos 1.9	Sild 1.8
**Female**	18	Clinically treated as IPAH. Query 1' Pulmonary veno-occlusive disease and/or pulmonary capillary hemangiomatosis	95	39.6	Epo 3.5	Bos 3.7	Sild 3.7
**Female**	35	IPAH	-	-	Iloprost 1.4	Bos 1.4	no
**Male**	43	IPAH	50	-	-	-	-

ASD, atrial septal defect; Bos, bosentan; CHD, congenital heart disease Epo, epoprostenol; ERA, endothelin receptor antagonist; IPAH, idiopathic PAH; mPAP, mean arterial pressure; PDE5i, phosphodiesterase type 5 inhibitor; PVRI, pulmonary vascular resistance index; Sild, sildenafil; VSD, ventricular septal defect

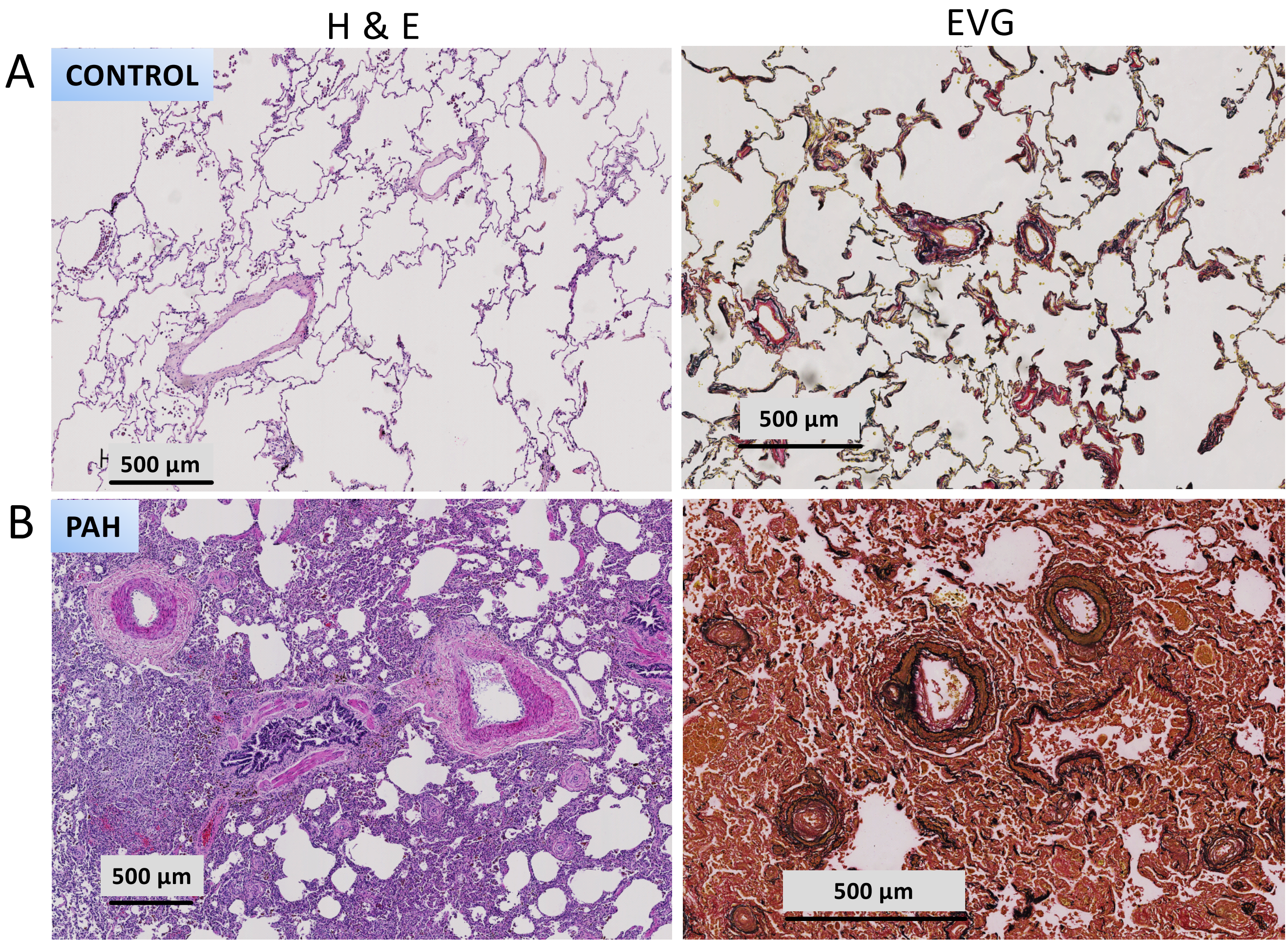

Figure S1. Gross pathological changes in the lungs of patients with pulmonary arterial hypertension (PAH). Immunohistochemical staining in 10 µm sections of lung tissue from controls (A) or patients with PAH (B) is shown. In the left panels, hematoxylin and eosin (H&E) staining is shown, where nuclei stain blue/purple while cytoplasm and muscle stain a purplish red. In the right panels, Van Gieson (EVG) staining shows collagen in red, elastic fibres and nuclei in black and other tissue elements in yellow. Scale bars are as indicated.

Table S2. Anti-proliferative effects of treprostinil in the absence and presence of IP and EP_2 prostanoid receptor antagonists; comparison with MRE-269 and butaprost.

**Treatment**	**pIC_{50}**	**I_{Max} (% inhibition)**
Treprostinil	7.96 ± 0.21 (n=6)	82.8 ± 3.6% (n=6)
Treprostinil + RO1138452	7.41 ± 0.45 (n=6)	77.2 ± 5.3% (n=6)
Treprostinil + PF-04418948	6.13 ± 0.34 (n=6)*#	86.3 ± 6.9% (n=6)
Treprostinil + PF-04418948 + RO1138452	5.5 ± 0.40 (n=5)*#	85.8 ± 3.7% (n=5)
MRE-269	8.36 ± 0.31 (n=5)	55.2 ± 3.9% (n=5)*
Butaprost	8.30 ± 0.76 (n=5)	65.6 ± 4.3% (n=5)*

To allow appropriate pharmacological statistical analysis, data are expressed in this table as mean pIC_{50} (negative log of IC_{50}, the concentration reducing proliferation by 50%) and I_{Max} values (maximal inhibition of cell proliferation). These parameters have been extrapolated for statistical comparison from sigmoidal (variable slope) fitting of individual concentration-response curves in each drug group using cell isolates of human pulmonary smooth muscle cells from the same group of pulmonary hypertensive patients. RO1138452 is an IP receptor antagonist and PF-04418948 is an EP_2 antagonist both of which were applied at 1µM. *$P<0.05$, when compared to treprostinil alone and $^{\#}P<0.05$, when compared to treprostinil in the presence of RO1138452 (one way ANOVA with Newman-Keuls multiple comparison test).

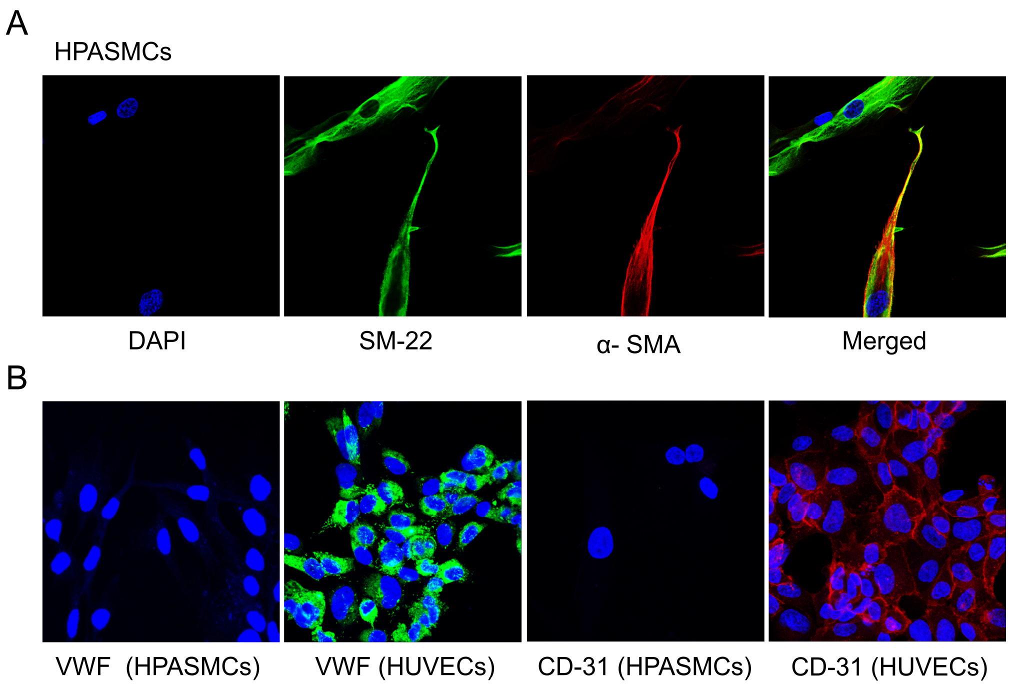

Figure S2: Confocal images of human pulmonary arterial cells derived from a PAH patient showing staining for classical smooth muscle but not endothelial cell markers. (A) Immunocytochemical staining with the nuclear stain DAPI and antibodies directed against smooth muscle markers, α-smooth muscle actin (α-SMA; red) and SM-22 (green) in human pulmonary arterial smooth muscle cells (HPASMCs) from a PAH patient. Images are taken from the same field of view, and staining of the individual markers merged (far right panel), showing α-SMA and SM-22 staining in the same cells and co-localised (yellow). (B) Lack of staining of the classical endothelial cell markers, CD-31 and von Willebrand factor in HPASMCs stained with DAPI, shown alongside positive antibody controls in human umbilical vein endothelial cells (HUVECs). Not shown is negative staining of α-SMA and SM-22 in endothelial cells.

Discussion: In HPASMCs from PAH patients, we observed co-localisation of α-SMA and SM-22, both classical smooth muscle cells markers but no staining of the endothelial cell markers, strongly suggesting cells are smooth muscle in origin. While α-SMA does not routinely stain endothelial cells or fibroblasts, it does however stain subpopulations of myofibroblasts [1], while SM-22 is not a widely recognised marker of this cell type. Interestingly, myofibroblasts have recently been shown to display simultaneous expression of von Willebrand factor (vWF) and α-smooth muscle actin in the subendothelial space of small and medium-sized arterioles in scleroderma but not control patients [2] and also in myofibroblasts transformed from endothelial cells [1]. This might be considered as further evidence for the relative purity of our smooth muscle cells from control and PAH patients, which do not stain for vWF.

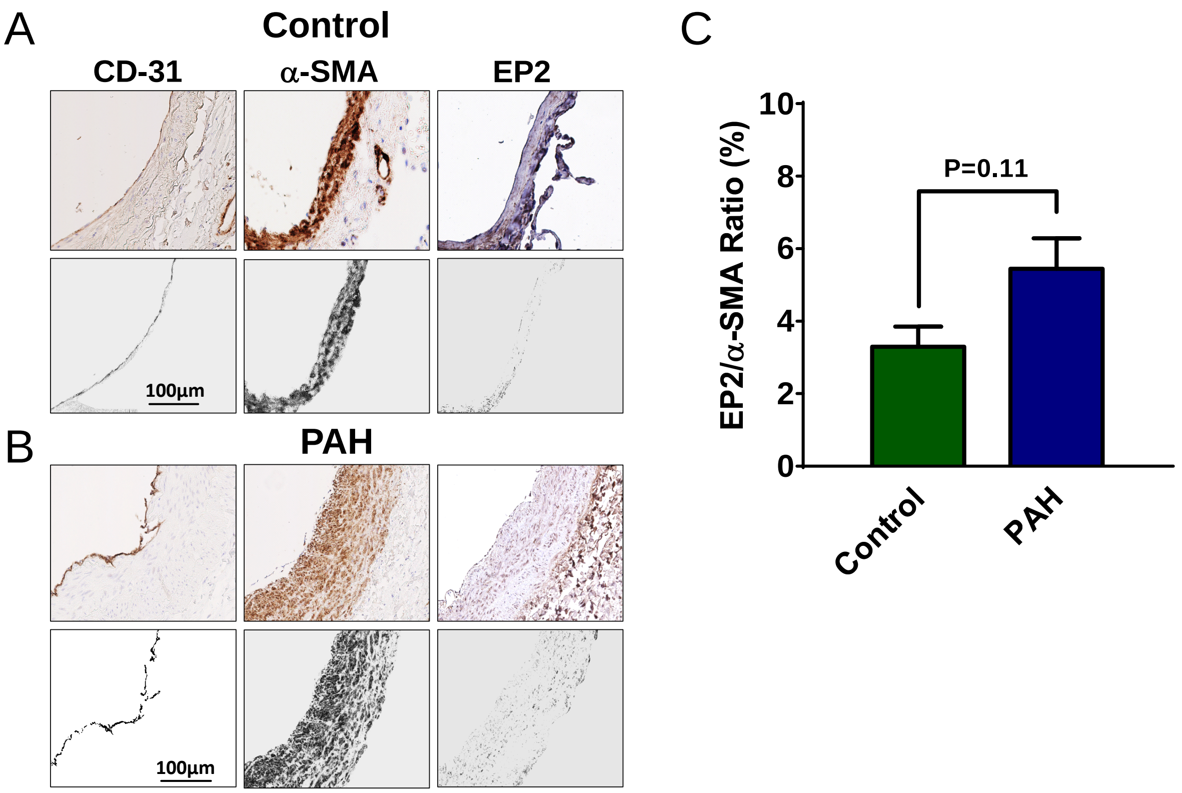

Figure S3. EP$_2$ receptor expression in large human pulmonary arteries.

Immunohistochemical staining was carried out in 10 µm serial sections of human lung tissue from a control patient (A) or a patient with pulmonary arterial hypertension (PAH) (B). Antibody staining for the endothelial cell marker, CD-31, the smooth muscle marker, α-smooth muscle actin (α-SMA) and the prostanoid EP$_2$ receptor (EP2) was visualised by diaminobenzidine (brown) in sections counterstained with haematoxylin. Below each tissue section, is the digitised image for individual markers, quantified using the colour threshold function in ImageJ as per methods. For clarity adventitial staining has been excluded from the digitised images. Data in C are from 6-12 different arteries from up to four patient isolates per group.

References from discussion of figure S2:

1. Wermuth PJ, Li Z, Mendoza FA, Jimenez SA. Stimulation of transforming growth factor-β1-induced endothelial-to-mesenchymal transition and tissue fibrosis by endothelin-1 (ET-1): A novel profibrotic effect of ET-1. *PLoS One*. 2016; **11**(9):e0161988.
2. Mendoza FA, Piera-Velazquez S, Farber JL, Feghali-Bostwick C, Jiménez SA. Endothelial cells expressing endothelial and mesenchymal cell gene products in lung tissue from patients with systemic sclerosis-associated interstitial lung disease. *Arthritis Rheumatol.* 2016; **68**(1):210-217.
